# The relationship between patient safety culture and patient safety competency with adverse events: a multicenter cross-sectional study

**DOI:** 10.1186/s12912-022-01076-w

**Published:** 2022-11-01

**Authors:** Asal Hafezi, Atye Babaii, Bahman Aghaie, Mohammad Abbasinia

**Affiliations:** Department of Medical-Surgical Nursing, Faculty of Nursing, Qom University of Medical Science, Qom, Iran

**Keywords:** Nursing, Patient safety, Adverse events, Safety management

## Abstract

**Background:**

Patient safety culture and patient safety competency could be associated with adverse events (AEs). This study aimed to investigate the associations between nurses’ perceptions of patient safety culture, patient safety competency, and AEs.

**Methods:**

A cross-sectional study was carried out among 338 nurses employed in three university hospitals in Qom, Iran between 17 August 2021 and 12 November 2021. Data were collected using three questionnaires: patient safety culture, patient safety competency, and AEs. Data were analyzed using SPSS-21 software. A multiple logistic regression model was used to analyze the data.

**Results:**

The results of this study showed that medication errors were significantly associated with “frequency of events reported” (OR = 0.706, *P* = 0.012), “supervisor/manager expectations and actions promoting patient safety” (OR = 0.733, *P* = 0.048), and “management support for patient safety” (OR = 0.755, *P* = 0.012). Pressure ulcers were significantly associated with “supervisor/manager expectations and actions promoting patient safety” (OR = 0.729, *P* = 0.039), “handoffs and transition” (OR = 0.707, *P* = 0.034), and “comfort speaking up about patient safety” (OR = 0.614, *P* = 0.016). Falls were significantly associated with “teamwork within units” (OR = 0.735, *P* = 0.031), “feedback and communication about error” (OR = 0.756, *P* = 0.046), and “handoffs and transition” (OR = 0.660, *P* = 0.012). The use of restraints for ≥8 hr. was significantly associated with “management support for patient safety” (OR = 0.701, *P* = 0.021).

**Conclusions:**

According to the results of this study, AEs are associated with some dimensions of patient safety culture and patient safety competency. Further research is needed to confirm these findings and identify interventions to reduce the occurrence of AEs.

## Background

In the health care system, AEs seriously affect patient safety and quality of care. AEs occur while providing care to the patient and could have negative consequences [1]. Despite many medical advances in treatment and diagnosis, AEs are still a challenge for healthcare workers and patients [2]. According to the World Health Organization (WHO), AEs is likely one of the 300 leading causes of death in the world [2]. According to a recent national study in 2021, the prevalence of AEs among nurses in Iran was 10 to 80% [3]. Another study in Iran showed that 29.1% of nurses had experienced at least one AEs within the last 6 months [4]. Most AEs do not occur due to a healthcare provider’s performance and are associated with defects in healthcare systems or care processes. These defects could lead health care providers to make mistakes [5]. Therefore, blaming individuals or punitive behavior cannot reduce AEs. Instead, focusing on the systems that allow errors to occur is seen as an important initiative to improve patient safety [6].

According to the International Council of Nursing (ICN), patient safety is the basis of quality care [7]. Therefore, improving the patient safety culture [8] and patient safety competency [9] are important strategies that health system managers have considered to improve the quality of nursing care. Safety culture has been described as individual and group values, perceptions, attitudes, competencies, and patterns of behavior that determine the commitment and skills of healthcare organizations [10]. Patient safety competence is also defined as the ability of healthcare professionals to minimize patient harm through individual performance [11]. In health care organizations, approaches to improving patient safety culture include communication based on mutual trust, good information flow, a shared understanding of the importance of patient safety, organizational learning, management and leadership commitment, and the adoption of a nonpunitive response to error reporting [12]. In the Iranian healthcare system, hospital accreditation and clinical governance have been introduced to improve the quality of care and patient safety [13]. However, some studies in Iran have shown that the patient safety culture is not at an ideal level and needs serious improvement [14, 15].

Nurses are a crucial part of any healthcare system. They have the most direct interaction with patients of any healthcare professional. Therefore, improving nurses’ ability to maintain patient safety is essential to the success of any comprehensive patient safety strategy [16]. Considering the high prevalence of AEs and the huge burden of their costs on the healthcare systems, nurses’ awareness of the patient safety culture is essential to improving patient safety and quality of care [17]. Various studies have assessed the associations between nurses’ perceived patient safety culture, patient safety competency, and AEs. Kakemam showed that nurses who scored higher in teamwork experienced fewer AEs [18]. Ravaghi et al. also argued that the incidence of medical errors is lower in hospitals with a better patient safety culture [19]. Kakemam et al. also indicated that nurses who had a higher score in the patient safety culture experienced fewer AEs [20]. However, DiCuccio discussed that limited studies reported a statistically significant correlation between patient safety culture and patient outcomes [21]. Choo and Choi assert that of the 10 specific aspects of patient safety culture, only the higher mean score of teamwork was associated with higher nurses’ safety competence [22]. Lee et al. claimed that there is no significant difference between patient safety scores and the cultural competencies of nursing students [11]. Han et al. showed that of the 10 dimensions of patient safety culture, only higher mean scores of open communication and teamwork were related to the lower AEs, and there was no significant relationship between the other dimensions of patient safety culture and the incidence of AEs [23].

As shown, some studies reported that patient safety culture and teamwork is an important factor in reducing the occurrence of AEs. However, according to the results of some other studies, the high level of only some dimensions of the patient safety culture in nurses is related to higher patient safety. Most studies have also estimated the frequency of AEs at the level of the single units and based on reporting systems. Therefore, considering the importance of AEs and their relationship with patient safety, this study aims to assess the associations between nurses’ perceived patient safety culture, patient safety competency, and AEs. Our research is based on the hypothesis that a higher level of nurses’ perception of patient safety culture and patient safety competency would be associated with lowered occurrence of AEs.

## Methods

### Study design

This cross-sectional study was conducted between 17 August 2021 and 12 November 2021.

### Setting and participants

The participants included nurses working in the three university hospitals in Qom, Iran. We selected the three biggest training and referral hospitals by the number of staff and beds. Three authors performed parallel sampling in three hospitals. The inclusion criteria included working full-time, having more than 3 year’s work experience, working in the clinical departments, and having consent to participate in the study. Random sampling was used to select the nurses. The sample size was calculated according to the results of an study conducted by Han et al. [23]. According to the results of Han et al., a type I error probability of 0.05 and a power of 0.80, the sample size was determined to be 330 nurses. However, for compensating probable attrition, we recruited 380 samples.

### Instruments

#### Demographic characteristics

Demographic characteristics of nurses (including gender, age, education, work experience, and workplace) were collected using a checklist.

#### Patient safety culture questionnaire

We measured patient safety culture using the Hospital Survey on Patient Safety Culture (HSOPSC) questionnaire developed by the Agency for Healthcare Research and Quality [24]. This questionnaire includes 42 items covering 12 dimensions. These dimensions are shown in Table 2. Responses are provided using a 5-point Likert-type scale of frequency, ranging from ‘never’ to ‘always’, or agreement, ranging from ‘strongly disagree’ to ‘strongly agree’. The mean scores are calculated for each dimension. Higher scores indicate a higher safety culture and vice versa. The Cronbach’s alpha coefficient of 0.57–0.80 was calculated for the Persian version of this questionnaire [25]. The Cronbach’s alpha coefficient was 0.74–0.84 in this study.

#### Patient safety competency questionnaire

Patient safety competency was measured using the Health Professional Education in Patient Safety Survey [26]. This questionnaire includes 38 items covering nine dimensions. These dimensions are shown in Table 2. Responses are provided using a 5-point Likert-type scale ranging from ‘strongly agree’ to ‘strongly disagree’. Higher scores indicate greater competency in maintaining patient safety. The Cronbach’s alpha coefficient of 0.73–0.86 was calculated for the Persian version of this questionnaire [27]. The Cronbach’s alpha coefficient was 0.85–0.92 in this study.

#### Adverse event

In this study, we selected the following four AEs that commonly occur in hospitals. These AEs included medication errors, pressure ulcers, falls, and use of physical limitation for more than 8 hours [28]. The rate of all AEs that nurses perceived over the last year was obtained using a 7-point Likert-type scale ranging from ‘not at all’ to ‘every day’ (6). The internal consistency reliability of this questionnaire estimate using Cronbach’s alpha was 0.789.

### Data collection

All nurses, were informed about the purpose of the study, voluntary participation, and the confidentiality of information. They filled the questionnaires in their resting room. A total of 380 questionnaires were distributed. However, 42 of the questionnaires were not returned and therefore were excluded from the analysis.

### Data analysis

Data were analyzed using SPSS 21 software (SPSS, Inc., Chicago, Illinois). Descriptive statistics were presented to describe the participants’ demographic characteristics, and their perception of patient safety culture, patient safety competency and AEs. We dichotomized the responses of AEs by coding ‘never’ as ‘never happened = 0’ and the rest of the responses as ‘had happened = 1’ based on a previous study [20]. Then, we used multiple logistic regression models to assess the relationship between AEs and dimensions of patient safety culture and patient safety competency alongside the control of all nurses’ demographic variables. The significance level was considered 0.05.

## Results

### Demographic characteristics

Out of 380 distributed questionnaires, 338 questionnaires were returned (response rate = 89%). The nurses’ demographic characteristics are presented in Table 1 Among the 338 nurses, 61.8% were women, with an average age of 32.12 years (SD 6.97; range 25–53). They were clinically experienced in nursing for 8.58 years on average (SD 6.89; range 3–25 years). The majority of nurses had a bachelor’s degree (89%). Regarding workplace wards where they worked, 41% worked in the intensive care units.

**Table 1:** Demographic characteristics of nurses (*n* = 338)

Variables	n (%)
Gender	
Male	129 (38.2)
Female	209 (61.8)
Education	
Bachelor's degree	301 (89)
Master's degree	37 (11)
Workplace	
Internal medicine ward	111 (33)
Surgical medicine ward	89 (26)
Intensive care unit	138 (41)

### Patient safety culture and patient safety competency

Table 2 demonstrates the status of patient safety culture and patient safety competency among nurses. The mean total score of patient safety culture was 3.08 ± 0.49, and ranged from 2.53 ± 0.79 (staffing) to 3.55 ± 0.69 (handoffs and transition). Also, the mean total score of patient safety competency was 3.84 ± 0.44, and ranged from 2.83 ± 0.45 (comfort speaking up about patient safety) to 4.14 ± 0.66 (managing safety risks).

**Table 2:** Descriptive statistics of the hospital survey on patient safety culture and health professional education in patient safety survey

Dimension	Mean ± SD
**Hospital Survey on Patient Safety Culture**	**3.08 ± 0.49**
Frequency of events reported	3.46 ± 0.87
Overall perceptions of patient safety	3.37 ± 0.71
Supervisor/manager expectations and actions promoting patient safety	3.12 ± 0.75
Organizational learning-continuous improvement	3.24 ± 0.84
Teamwork within units	3.49 ± 0.81
Communication openness	2.98 ± 0.76
Feedback and communication about error	3.14 ± 0.83
Nonpunitive response to error	2.58 ± 0.88
Staffing	2.53 ± 0.79
Management support for patient safety	2.94 ± 0.76
Teamwork across units	3.06 ± 0.69
Handoffs and transition	3.55 ± 0.69
**Health Professional Education in Patient Safety Survey**	**3.84 ± 0.44**
Clinical safety	3.63 ± 0.39
Working in teams with other health professionals	3.96 ± 0.72
Communicating effectively	4.1 ± 0.76
Managing safety risks	4.14 ± 0.66
Understanding human and environmental factors	4.04 ± 0.68
Recognize, respond to and disclose adverse events and close calls	3.95 ± 0.70
Culture of safety	4.04 ± 0.68
How broader patient safety issues are addressed in health professional education	3.23 ± 0.82
Comfort speaking up about patient safety	2.83 ± 0.45

### Frequency of AEs

Table 3 reports the prevalence of AEs. The majority of participants reported that four AEs happened “several times a year.” A few participants stated that AEs happened “every day,” “several times a week,” and “once a week.” Only 1.8% of nurses reported that the restraint use ≥8 h occurred “every day.” None of the nurses reported that medication errors, pressure ulcers, and falls occurred every day.

**Table 3:** Frequency of adverse events in the past year among nurses (*n* =338)

Adverse events	Never happened, N (%)	Several times a year, N (%)	Once a month or less, N (%)	Several times a month, N (%)	Once a week, N (%)	Several times a week, N (%)	Every day, N (%)
**Medication errors**	153 (46.6)	128 (39)	28 (8.5)	16 (4.9)	1 (.3)	2 (.6)	0 (0)
**Pressure Ulcers**	128 (39.1)	155 (47.4)	25 (7.6)	13 (4)	4 (1.2)	2 (.6)	0 (0)
**Falls**	105 (32.1)	196 (59.9)	24 (7.3)	2 (.6)	0 (0)	0 (0)	0 (0)
**Restraint Use ≥8 hr**	120 (36.7)	135 (41.3)	32 (9.8)	20 (6.1)	2 (.6)	12 (3.7)	6 (1.8)

### Association between patient safety culture and AEs

The results of multiple logistic regression models are presented in Table 4. Only the variables that were significant in the model are put in this table. After controlling the confounding effects of demographic factors, the results did not change significantly. The results of this study showed that medication errors were significantly associated with “frequency of events reported” (OR = 0.706), “supervisor/manager expectations and actions promoting patient safety” (OR = 0.733), and “management support for patient safety” (OR = 0.755). The odds of experiencing medication errors increased by 34, 31, and 28% with each one-unit increase in scores for “frequency of events reported,” “supervisor/manager expectations and actions promoting patient safety,” and “management support for patient safety” respectively. Pressure ulcers were significantly associated with “supervisor/manager expectations and actions promoting patient safety” (OR = 0.729) and “handoffs and transition” (OR = 0.707). The odds of experiencing pressure ulcers decreased by 31 and 34% with each one-unit increase in scores for “supervisor/manager expectations and actions promoting patient safety” and “handoffs and transition,” respectively. Falls were significantly associated with “teamwork within units” (OR = 0.735), “feedback and communication about error” (OR = 0.756), and “handoffs and transition” (OR = 0.660). The odds of experiencing falls decreased by 30, 28, and 41% with each one-unit increase in scores for “teamwork within units,” “feedback and communication about the error,” and “handoffs and transition,” respectively. The use of restraints for ≥8 hr. was significantly associated with “management support for patient safety” (OR = 0.701). The odds of experiencing using restraints for ≥8 hr. decreased by 35% for each one-unit increase in scores for “management support for patient safety.”

**Table 4:** Multiple logistic regression results of the relationship between dimensions of patient safety culture and patient safety competency with the adverse events

Variables	OR (95% CI)	p
**Medication errors**		
Frequency of events reported	.706 (.539, .935)	.012^*^
Supervisor/manager expectations and actions promoting patient safety	.733 (.539, .997)	.048^*^
Management support for patient safety	.755 (.560, 1.019)	.012^*^
**Pressure ulcers**		
Supervisor/manager expectations and actions promoting patient safety	.729 (.540, 0.984)	.039^*^
Handoffs and transition	.707 (.514, .974)	.034^*^
Comfort speaking up about patient safety	.614 (.414, .913)	.016^*^
**Falls**		
Teamwork within units	.735 (.556, .972)	.031^*^
Feedback and communication about error	.756 (.575, .994)	.046^*^
Handoffs and transition	.660 (.477,.913)	.012^*^
**Use of restraints for ≥8 hr**		
Management support for patient safety	.701 (.519, 0.947)	.021^*^

*P < 0.05

### Association between patient safety competency and AEs

In the multiple regression models that controlled nurses’ demographic and organizational characteristics, a significant association was observed between pressure ulcers and “comfort speaking up about patient safety” (OR = 0.614). The odds of experiencing medication errors decreased by 48% for each one-unit increase in scores for “comfort speaking up about patient safety” (Table 4).

## Discussion

This study examined the associations between nurses’ perceptions of patient safety culture, patient safety competency, and AEs. Regarding patient safety culture, staffing and nonpunitive response to error were the lowest among all dimensions. These findings were consistent with a previous study conducted in Iran (2022) which showed staffing and nonpunitive response to error received the lowest score [15]. Han et al. reported that the lowest score obtained from the nurses’ safety culture was related to staffing [23]. This finding is also consistent with the results of the study by Nie et al., in which the nurses mentioned that the most significant obstacle to maintaining the patient’s safety was fear of being punished following the report of AEs [29]. Reason and Hobbs declared learning culture, justice, and reporting as critical components of patient safety culture. Therefore, medical authorities should consider mistakes as valuable learning opportunities to improve the patient’s safety culture while improving the AEs reporting process. Institute of Medicine stated that in the organizations whose employees report their errors without any fear, they could learn from their errors and prevent repeating them. This institute of considers changing of patient safety culture as the greatest challenge in safer care approach. Therefore, healthcare organizations should assess their patient safety culture and develop comprehensive patient safety programs to increase detection of AEs, and redesign systems to reduce opportunities for error (8).

Regarding nine dimensions of patient safety competency, comfort speaking up about patient safety was found with the lowest score. According to the findings of this study, nurses’ competency in patient safety was at a moderate level. Cho and Choi also declared that nurses’ competency in performing safe clinical procedures for the patient is at a moderate level [22]. A previous study in Iran showed that patient safety competency among nurses is at a moderate level [30]. These findings indicate the importance of empowering nurses to perform safe clinical procedures to improve their safety competency.

Our findings showed that about 40% of nurses (39–59.9%) commit AEs several times a year, with falls as the most (59.9%) and medication errors (39%) as the least common. Kang et al. also reported a 35–60% rate for AEs. Kang declared a rate of 60.5 and 33.3% for medication errors and falls, respectively [31]. Kakemam et al. also reported 35.7 and 34.5% for medication errors and falls, respectively [20]. Accordingly, a significant percentage of hospitalized patients suffer from AEs, which indicates the importance of careful and systematic planning by health policymakers to prevent such events.

Based on the multiple logistic regression, the “frequency of events reported” predicted medication errors in the patient safety culture. The first step in reducing medication errors and improving patients’ immunity is studying the different types of AEs. In this way, we can better understand why AEs occur. Therefore, reporting and analyzing all AEs is essential for reducing medication errors [32]. However, despite the mandatory reporting of medication errors in the health care system, reporting fewer of these errors is a common challenge [33]. In this regard, Kakemam et al. also state systems thinking had a significant role in preventing the occurrence of AEs and improving the reporting of AEs [34]. Rodziewicz declared that reporting errors could reduce medication errors. He emphasizes that if health care providers report the circumstances in which AEs have occurred, managers can improve service delivery practices and reduce AEs [5]. According to these findings, establishing a systematic and comprehensive system for reporting errors and AEs seems crucial because it leads to identifying the types, nature, and causes of errors, by which the processes to reduce or eliminate similar errors can be planned [35]. In addition, based on our results, supervisor/manager expectations and actions promoting patient safety can predict pressure ulcers. By providing frequent feedback to nurses, nursing managers can improve nurses’ skills and ability to solve problems and prevent AEs [36].

According to the results of our study, higher mean scores for “supervisor/manager expectations and actions promoting patient safety” was significantly correlated with medication errors and pressure ulcers. Also, “management support for patient safety” predicted medication errors and the use of restraints for ≥8 hr. Leadership and management styles of healthcare organizations can effectively reduce or increase AEs [37]. Liukka et al. argued that nursing managers should motivate and empower health care providers to encourage them to find new ways to provide health services to prevent AEs and improve patient safety [38]. Nurmeksela et al. emphasized that managers of healthcare centers should improve the motivation and sense of security of healthcare providers by focusing on care delivery methods. They should adopt a leadership style that emphasizes safe and patient-centered care [39].

Also, higher mean scores for “teamwork within units” were significantly correlated with falls. Providing health care is complex, and it is impossible to ensure that all the patient receives the highest standard of care and is safe from all possible harm from complex treatments. Therefore, providing standard and safe care requires the high cooperation of health care providers [40]. In this regard, Weaver et al. Also emphasized that providing safe surgical care requires effective teamwork [41].

According to the results of this study, “feedback and communication about error” was significantly correlated with falls. Nursing managers prevent AEs by providing frequent feedback on errors and developing nurses’ problem-solving skills [36]. Providing feedback about reporting errors can encourage healthcare providers to report errors voluntarily regardless of their severity [42]. Error reporting can help health system managers identify the causes of errors and design correction programs to prevent the recurrence of errors and improve patient safety [43]. Woo and Avery found that nursing leaders could enhance patient safety by providing appropriate feedback on reported errors [44].

Furthermore, higher mean scores for “handoffs and transition” were significantly correlated with reduced occurrences of pressure ulcers and falls. The transition of care frequently occurs between care providers. Safe patient transfer requires the accurate transfer of patient information by care providers to new caregivers [45]. In line with the results of this study, Starmer et al. reported that a structured handoff communication program could reduce preventable AEs by up to 30% [10]. Lee also stated that the transition of appropriate information, responsibility, and accountability are essential to improve patient safety [46]. Therefore, standardized and structured handoffs and transitions can effectively promote patient safety [45].

Based on the multiple logistic regression, in patient safety competency, “comfort speaking up about patient safety” predicted pressure ulcers. “Speaking up” is defined as the discussion of health care professionals to improve the quality of care and patient safety after recognizing or being aware of health care providers’ risky or deficient actions [47]. Joint Commission discussed that up to 80% of the AEs occur due to miscommunications among professionals [48]. Nacioglu declared that awareness of the effective factors in empowering speaking skills in healthcare providers could help health managers to improve the quality and safety of health care in their organization [49].

### Study limitation

Our study had several limitations that should be considered when interpreting the results. Firstly, the cross-sectional design was used in this study. Further research, using other designs such as longitudinal and controlled studies, is needed to determine the effectiveness of patient safety culture and patient safety competency. Secondly, self-administered questionnaires were used to assess the patient’s safety culture, patient safety competency, and adverse effects. Therefore, an over-or underestimation of the results may occur.

## Conclusion

This study examined the relationship between patient safety culture and patient safety competency with the incidence of AEs. Our findings showed that nurses’ perception of patient safety culture and competency is associated with AEs. According to the results of our study, “frequency of events reported,” “supervisor/manager expectations and actions promoting patient safety,” “management support for patient safety,” “teamwork within units,” “feedback and communication about error,” “handoffs and transition,” and “comfort speaking up about patient safety” are associated with AEs.

In conclusion, hospital executives should have plans to improve patient safety. They should also provide appropriate feedback on reported errors and encourage nurses to frequency reporting the AEs, correctly handoffs and transition, speaking up about patient safety, and teamwork within units to prevent AEs. Further research is needed to confirm these findings and identify interventions to reduce the occurrence of AEs.

## Data Availability

All data generated or analyzed during this study is included in this article.

## Abbreviations

AEs: Adverse Events
ICN: International Council of Nursing
HSOPSC: Hospital Survey on Patient Safety Culture
